# At the Research Frontiers of Small GTPases

**DOI:** 10.3390/cells11233708

**Published:** 2022-11-22

**Authors:** Bor Luen Tang

**Affiliations:** Department of Biochemistry, Yong Loo Lin School of Medicine, National University of Singapore, Singapore 117596, Singapore; bchtbl@nus.edu.sg

**Keywords:** GTPase activating proteins (GAPs), guanine nucleotide exchange factors (GEFs), KRAS, Ras, Rho, small GTPases

## Abstract

Small GTPases act as molecular switches in regulating a myriad of cellular signaling, cytoskeletal dynamics, vesicular trafficking, and membrane/organelle transport processes. Here, I provide an editorial overview of papers collected in this Special Issue on the “Regulation and Function of Small GTPases 2.0”.

The Ras superfamily of small GTPases functions as signaling relays and regulatory consolidation nodes for diverse cellular physiological and pathophysiological pathways and processes. Cycling between their inactive GDP-bound form and effector-engaging, active GTP-bound form, small GTPases connect upstream events to downstream factors that ultimately impact cell proliferation, cellular architecture, cytoskeletal dynamics, membrane/organelle trafficking, and a myriad of other processes underlying cellular homeostasis. Studies that shed light on the mechanisms of regulation and action of small GTPases and their regulators have provided important insights into the inner workings of the cell, deepened our understanding of disease pathways, and revealed potential biomarkers and therapeutic targets. Given the vast and diverse literature on small GTPases, having a collection of articles providing overviews as well as an in-depth discussion on the latest discoveries would be helpful to researchers in the field and interested general readers.

This Special Issue of *Cells* on the “Regulation and Function of Small GTPases 2.0” comprises five review articles and an original research paper. Dautt-Castro and colleagues presented an interesting and valuable overview and analysis of small GTPase families in fungi, based on 56 different genomes from different phyla, in terms of phylogenetics, structure, function, as well as their roles in virulence and pathogenesis [1]. Other than mycologists, their carefully curated information would be very helpful to those working with the mammalian system wishing to obtain an overview of the advances made on relevant orthologues in fungi.

Han and colleagues reviewed the KRAS oncogene, its guanine nucleotide exchange factors (GEFs) and GTPase activating proteins (GAPs), and its downstream signaling components and narrated an interesting tale of cancer drug quest against KRAS mutants [2]. Despite being one of the most commonly mutated oncogenes found in approximately ~30% of human cancers, KRAS has been conventionally considered undruggable as it lacks classically useful drug-binding sites, particularly ones with a deep hydrophobic pocket. However, more recent advances have changed this gloomy outlook, particularly with the discovery of G12C inhibitors and sotorasib (AMG510) becoming the first FDA-approved KRAS-mutation-targeting cancer drug in 2021. The more recently discovered G12D inhibitors also appear promising.

Mosaddeghzadeh and Ahmadian reviewed the Rho family GTPases [3]. This key family of proteins that regulates cytoskeletal dynamics comprises 20 canonical Rho GTPases, which are regulated by three GDIs, 85 GEFs, and 66 GAPs, with the activated Rho family members, in turn, interacting with more than 70 downstream effectors. The authors provided a thorough yet concise summary of this elaborate molecular network, which would be a very useful reference source. On the other hand, Machin et al. focused on the emerging roles of Rho family members and Rho GEFs in glucose homeostasis [4]. This homeostatic regulation, which controls blood glucose levels, occurs in two major ways. A number of Rho GEFs participate in insulin-stimulated translocation of the glucose transporter GLUT4 to the plasma membrane, which promotes blood glucose uptake into skeletal muscle and adipose tissue. On the other hand, an overlapping set of Rho GEFs is involved in glucose-stimulated insulin secretion by pancreatic β-cells. The authors’ comprehensive coverage of molecular players and mechanisms provided a detailed picture of Rho GEF-dependent glucose homeostasis, which is critical to the understanding of (and thus of great interest to those working on) cellular and systemic defects underlying type 2 diabetes and metabolic syndrome.

Microtubules are polymers of α and β-tubulin, both of which can bind guanine nucleotides, as does γ-tubulin, which forms the cellular γ-tubulin meshwork. Although not considered a member of the Ras superfamily, β-tubulin hydrolyzes GTP during microtubule polymerization and undergoes GTPase-dependent switching between phases of growing and rapid shrinking, a phenomenon known as dynamic instability. Kristensson reviewed the GTP-binding domains of α-, β-, and γ-tubulin and elaborated on the functions of the γ-tubulin GTP-binding domain in the regulation of the γ-tubulin meshwork dynamics and cellular homeostasis [5]. In the only original research paper in the collection, Hampson and colleagues revealed the role of P-Rex1, a GEF that activates Rac small GTPases in response to G-protein-coupled receptors (GPCR) activation, in sphingosine 1-phosphate (S1P) signaling [6]. The authors showed, using PC12 cells that stably overexpressed the S1P receptor, that P-Rex1 is required for the S1P-stimulated activation of Rac1 and Akt.

The above collection of papers, albeit limited in scope, proves useful as reference sources for those embarking on work in the field and those with a relevant interest. In addition, advances continue to be made in uncovering the physiological and pathological activities of other small GTPases not featured in this collection, such as members of Rab and Arf GTPases, as well as Ran. Fortunately, readers can anticipate learning about these advances in small GTPases 3.0. 
